# Editorial: Complement and COVID-19 Disease

**DOI:** 10.3389/fimmu.2022.960809

**Published:** 2022-07-01

**Authors:** Zoltán Prohászka, Nicolas S. Merle

**Affiliations:** ^1^ Research Group for Immunology and Haematology, Semmelweis University- Eotvos Lorand Research Network (Office for Supported Research Groups), Department of Internal Medicine and Haematology, Semmelweis University, Budapest, Hungary; ^2^ Department of Internal Medicine and Haematology, Semmelweis University, Budapest, Hungary; ^3^ Complement and Inflammation Research Section (CIRS), National Heart, Lung, and Blood Institute (NHLBI), National Institutes of Health (NIH), Bethesda, MD, United States

**Keywords:** COVID-19, SARS – CoV – 2, complement system, thromboinflammation, kinin-kallikrein system

Since December 2019 and the first confirmed case of SARS-CoV-2 in Wuhan, China, the world has faced an unprecedent global health crisis. Within 2.5 years, more than six million and counting individuals have died from COVID-19 disease, and at least half a billion cases have been reported. The COVID-19 crisis has been answered with an unprecedent effort of the entire Scientific community that was accompanied by worldwide safety measures and next-generation of mRNA vaccines. By inducing the production of specific neutralizing antibodies against the spike protein of SARS-CoV-2 to prevent virus entry in targeted cells, large-scale vaccination dramatically helped reducing the quick propagation of the virus. Unfortunately, despite all those efforts, individuals may still suffer from many complications that would require oxygen supply and lead to death from SARS-CoV-2.

Early on in the pandemic, many co-morbidities and factors – such as age, genetics, pre-existing disease condition– were identified as increasing risks of severe COVID-19 disease and related death. While it helped improving patients’ management and saved many lives, deciphering the biological reaction leading patient’s complications is necessary to provide better care and prevent death. Evidences quickly pointed towards an inappropriate immune response as being, at least in part, responsible for COVID-19 complications. Indeed, many studies conducted throughout the pandemic led to the current understanding of a ‘bipolar’ immune response. If an early inflammation is beneficial to prevent virus entry and its spread throughout the body, it becomes detrimental for the host when the whole system is not properly controlled and shutdown. When happening, overactivation of innate immune system and the subsequent cytokine storm escalates to severe lung injuries and generalized inflammation as observed in Multisystem Inflammation Syndrome (MIS). Based on this understanding, clinical trials and drug-repurposing established that anti-inflammatory treatment (e.g. dexamethasone and tocilizumab) would help lower down escalation of the inflammatory response. However, further studies are still necessary to acquire deeper understanding of the mechanisms at play to develop better targeted therapies and improve our knowledge of SARS-CoV-2 virus.

The complement system is part of the innate immunity. It is implicated in many pathological processes, particularly in hemolytic disorders associated with thrombotic microangiopathy (TMA) where monoclonal anti-C5 therapy eculizumab has revolutionized life prognosis (1). This ancient cascade has established shared activation and regulation at least in part, with many systems, ranging from chemotaxis activities to intricate relationships with coagulation pathways and kallikrein-kinin system. In COVID-19, besides inflammation, thrombosis is a clinical feature shared among patients suffering from severe symptoms. Furthermore, the hijacking of ACE2 protein by SARS-CoV-2 virus to enter cells that impairs its function led to speculation of an imbalanced kallikrein-kinin system and was soon after demonstrated to participate in the thromboinflammation caused by COVID-19 (2, 3). Finally, a sustained inflammation and high levels of complement activation were found in SARS-CoV-2 infected patients observing an unusual long course of the disease with late complications, also known as long COVID-19. Altogether, these evidences suggested a key role of the complement system in the clinical complications faced by those patients.

In this Research Topic, we aimed to gather Original Research articles, Reviews and Case Reports which have improved our understanding about the relation between complement system and COVID-19 disease severity, long-COVID-19, and outcomes. A total of fourteen articles were published under this collection addressing how complement system is activated by SARS-CoV-2, but also how complement proteins could be used as biomarkers for COVID-19 disease.

Complement system impairment has been documented as directly or indirectly involved in pathophysiological mechanisms of many diseases, including autoimmune and infection diseases (4). Easy access to complement proteins by serology make them markers of choice to identify pathologies in patients. In more recent years, complement deposits and circulating complement protein levels have been increasingly reported as predictive markers in different disease courses (5–8). In a cohort of 128 patients with COVID-19, Sinkovits et al. described that overactivation and consumption of C3 correlated with other markers of inflammation – such as IL6 and CRP – and can be used as predictive marker of mortality of SARS-CoV-2 infection. This result echoed the meta-analysis performed on 19 studies and more than 3700 COVID-19 patients by Zinellu and Mangoni and determining C3 and C4 as potential predictive factors of COVID-19 severity and mortality at the peak of the disease. Moreover, the study from Senent et al. suggested that complement activation is linked to long-term COVID-19. By analyzing samples from a total of 50 patients over the course of 3 months, authors were able to correlate high levels of circulating C5a with the presence of long-term respiratory symptoms suggesting the use of C5a as a potential biomarker for long-term SARS-CoV-2 infection.

Considering the length and complexity of the inflammation caused by COVID-19, complement system activation is likely driven by many different signals leading to activation of the three pathways rather than an exclusive one. Nonetheless, it would be expected that implication of each pathway to be time- and context-dependent.

For instance, lectin pathway importance in COVID-19 disease was challenged by Panteleimon Charitos et al. but authors rather found an association between classical and alternative pathways activities and critically ill patients. Noteworthy, SARS-CoV-2 receptor-binding domain drives IgG1 and IgG3 subclasses response (9–11). Considering their ability to bind C1q and to activate the classical pathway, Jarlhelt et al. demonstrated that IgG levels and disease severity directly correlate with classical pathway activation, indicating that elevated IgG levels and/or severe disease might be associated with prominent complement activation during viral infection.

However, by analyzing deposition of complement proteins in lungs and kidneys from patients who died from SARS-CoV-2 infection, Niederreiter et al. showed significant increase of MASP-2, C3d and C5b-9, suggesting that the lectin pathway is involved in worsening systemic inflammatory response. This was further supported by a study on a cohort of 74 hospitalized patients due to COVID-19, where Defendi et al. revealed that high levels of lectin pathway activation constituted the higher proportion of patients who required oxygen support or ICU care and died. Besides, the *in vitro* investigation conducted by Ali et al. incriminates the lectin pathway for inducing C3b deposition on cell surface and corroborated the encouraging performance of MASP-2 inhibitor Narsoplimabin a clinical trial conducted on severe COVID-19 patients (12).

Crosstalk between intravascular cascades like complement, coagulation and kallikrein-kinin systems can collectively contribute to cytokine storm, general inflammation and acute respiratory distress syndrome observed in SARS-CoV-2 infection. Savitt et al. showed that SARS-CoV-2 proteins – Envelope, Spike, Nucleocapside and Membrane proteins – can directly activate the classical complement, the coagulation and the kallikrein-kinin systems by binding to C1q, FXII and high molecular weight kininogen, respectively. Furthermore, they showed that viral proteins turn globular C1q Receptor as a platform for complement and kallikrein-kinin pathways, indicating a direct cross-reactivity between the 2 systems.

Pre-existing disease may be an aggravating factor of COVID-19 disease. Accordingly, Peerschke et al. established that thromboinflammation as evidenced by increased plasma D-dimer levels in cancer patients was associated with elevated complement activation. This was further linked with increased mortality in the setting of COVID-19, emphasizing the potential of complement system as a predictive marker in COVID-19 survival.

Thromboinflammation being a key feature associated with COVID-19 death, it is fair to assume that pre-existing hemolytic disease condition would inflate SARS-CoV-2 infection and worsen patients’ complications. This hypothesis is particularly relevant here, considering that complement is usually involved in organ injuries occurring in hemolytic disorders. In this context, vaccination would be critical to prevent exacerbation of pre-existing hemolytic sensitivity. Fattizzo et al. reported 4 cases – supported by a review of the literature –with complement-mediated hemolytic anemia pre-condition who experienced hemolytic event within 10 days upon infection with SARS-CoV-2. Overall, patients were experiencing more complications and more fatalities compared to groups that received vaccination. Moreover, other therapies like complement inhibitors are especially to be considered in these cases to prevent death. Boudhabhay et al. reported a case of MIS associated with renal Thrombotic Micro-Angiopathy (TMA) and Acute Kidney Injury (AKI) in a 46-year-old patient with hypertension and obesity personal history. In this case report, authors successfully resolved AKI and dramatically improved renal function upon treatment with eculizumab, strengthening the therapeutic potential of anti-complement therapies in COVID-19 patients.

Interestingly, Ali et al. described a cohort of 217 patients with severe COVID-19 disease in which defect in circulating complement proteins induced by the infection seems to predispose to bacterial infections, most frequently *K. pneumoniae*.

Overall, clinical and basic studies published in this Research Topic and elsewhere revealed that complement system is a key partner of the thromboinflammatory reaction occurring in COVID-19-associated organ injuries. Nilsson et al. reviewed the complexity of the inflammatory response and the intricate relationships between the complement, the coagulation and the kallikrein-kinin systems. Authors elegantly summarized how life-threatening COVID-19 ARDS is associated with a strong activation of the intravascular innate immune system (IIIS), and with the recognition of this link future clinical trials involving the use of complement inhibitors may be fueled with observational data.

Deciphering the mechanisms and understanding the subtilities of the immune response is a long way to go. However, we hope that the variety of articles published in this Research Topic have provided insights on the link between complement, inflammation, and COVID-19 disease, with promising outcomes and future perspectives. As Editors, we would like to thank all the contributing authors and the people in Frontiers in Immunology for their contribution and excellent editing support.

## Author Contributions

All authors listed have made a substantial, direct, and intellectual contribution to the work and approved it for publication.

## Conflict of Interest

The authors declare that the research was conducted in the absence of any commercial or financial relationships that could be construed as a potential conflict of interest.

## Publisher’s Note

All claims expressed in this article are solely those of the authors and do not necessarily represent those of their affiliated organizations, or those of the publisher, the editors and the reviewers. Any product that may be evaluated in this article, or claim that may be made by its manufacturer, is not guaranteed or endorsed by the publisher.
